# Turning crisis into potential: Leveraging the skills of forced Ukrainian migrant women in Poland

**DOI:** 10.1371/journal.pone.0338655

**Published:** 2026-01-23

**Authors:** José Enrique Castillo-Sánchez, Paul Phumpiu Chang, Agnieszka Olter-Castillo

**Affiliations:** 1 Faculty of Economic Sciences, University of Warsaw, Warsaw, Poland; 2 Pontifical Catholic University of Peru, Lima, Peru; 3 The World Bank, Washington, D.C., United States of America; 4 Faculty of Political Science and International Studies, University of Warsaw, Warsaw, Poland; Wrocław University of Science and Technology, POLAND

## Abstract

The 2022 Russian invasion of Ukraine triggered a large influx of highly skilled Ukrainian women into Poland, presenting both challenges and opportunities for their effective economic integration. This study employs network analysis on firm-level data from the Orbis database to identify 32 key industries – including food manufacturing, textiles, business services, wholesale trade, and healthcare – where Ukrainian managerial talent can enhance Poland’s industry space. Guided by a conceptual framework of three mechanisms for integration – (1) Matching and Complementarities, (2) Diversification via Relatedness, and (3) Gendered Sectoral Constraints – we examine how sectoral skill alignment interacts with gendered barriers to shape integration outcomes. By examining industry specialisation patterns and gender dynamics, we assess how leveraging the expertise of these skilled migrant women can drive economic diversification, fill labour shortages, and stimulate innovation. Our findings underscore that these women can strengthen market competitiveness in industries where Poland has a specialisation advantage and contribute to economic diversification in emerging industries – provided that gender-specific barriers are addressed. This study contributes to the broader discourse on migration, gender, and industrial policy, offering an empirical framework for other host countries seeking to transform skilled refugee inflows into engines of economic growth. The findings emphasise the necessity of evidence-based policies that align skilled migration integration strategies with industrial and gender equity objectives to maximise economic benefits.

## Introduction

Understanding international migration remains a critical challenge in the 21st century [1], particularly in the context of forced displacement due to conflicts. The Russian invasion of Ukraine in 2022 triggered a mass exodus, marking Europe’s most significant humanitarian crisis since the mid-20th century. Before the war, Poland was already home to over 1 million Ukrainians. However, within weeks of the conflict, an additional 1.5 million forced migrants arrived, over 80% of whom were women of working age, with 50% holding higher education qualifications [2]. This unprecedented influx presents not only a humanitarian challenge but also an economic opportunity to integrate a skilled labour force into the Polish economy. Indeed, evidence from previous refugee inflows in Europe suggests that swift labour market inclusion of refugees can yield net economic gains for host countries [3]. Achieving such gains requires comprehensive integration strategies, which, as Ager and Strang [4] outline, must address both structural and socio-cultural dimensions. In response to this influx, Poland implemented short-term measures such as the Temporary Protection Directive, granting Ukrainian war refugees nearly unrestricted access to the labour market, while also recognising the importance of a long-term strategic approach to ensure their effective labour market integration [5].

It is important to consider that migrant selectivity patterns indicate that Ukrainian refugees in Poland may not fully represent Ukraine’s pre-war labour force composition. Highly skilled professionals often gravitate towards destinations with more developed managerial job markets, such as Germany and Austria, leading to a potential underrepresentation of this group in Poland. Thus, understanding where and how these women can best integrate into Poland’s economy requires a data-driven, industry-targeted approach.

This study addresses these challenges through an economic and migration policy lens, exploring which sectors can maximise the integration of highly skilled Ukrainian women while also enhancing Poland’s industrial competitiveness. Specifically, it investigates the following key questions:

1. Which Polish industries exhibit specialisation gaps that Ukrainian women’s expertise can fill?
2. Can integrating skilled Ukrainian women drive economic diversification through new industries or enhance competitiveness in existing ones?

To answer these questions, we employ a novel approach by combining network analysis with industry specialisation metrics to identify sectors where the knowledge and skills of Ukrainian professional women can have the most impact. Using firm-level data from the Orbis global database, we construct a benchmark industry network using data from major European host economies and Ukraine, and then map Poland’s position relative to that benchmark. This benchmarking is methodological scaffolding; our inferential focus is Poland. Comparisons to Ukraine are used to identify industries in which Ukrainian women held pre-war managerial roles and specialisations that are most likely to translate into Poland’s context. This design allows us to assess which industries have the strongest potential for absorbing Ukrainian managerial expertise and where their integration could yield the greatest economic spillovers.

Conceptually, we frame integration outcomes through three interrelated Mechanisms: Matching and Complementarities (M1) – placing skills in industries where Poland is already specialised and can absorb them efficiently; Diversification via Relatedness (M2) – branching into industries closely linked to existing strengths, fostering innovation and resilience; Gendered Constraints (M3) – structural and social factors that condition the utilisation of women’s skills in the labour market. These mechanisms jointly provide an integration oriented framework that captures both the economic potential of skilled refugee women and the gender-specific barriers that influence whether this potential is realised. Such industry-focused analyses respond to the call in migration economics, made by Bahar and Rapoport [6], to move beyond aggregate employment statistics and instead identify how migrant human capital aligns with host-country comparative advantages.

By situating our analysis within the broader migration and labour market integration literature, we not only contribute to a growing body of research on skilled migration, gendered labour dynamics, and industry specialisation but also place a strong emphasis on policy research. Our study highlights the critical role of evidence-based policymaking in shaping effective labour market integration strategies, fostering economic competitiveness, and ensuring that skilled migration is leveraged as a tool for industrial innovation and long-term growth. This perspective aligns with the work of Kancs and Lecca [7], who emphasise that integration outcomes are shaped not only by individual capabilities but also by host-country institutional and industrial structures.

While much of the existing literature on refugee integration focuses on low-skilled employment [8–10], this study shifts the focus to strategic labour market integration for highly skilled migrants. By bridging migration research and industrial policy, this study demonstrates how skilled refugees can reshape a host country’s industry landscape – a phenomenon observed historically with other migrant groups [11]. Our findings underscore the importance of policy-driven market integration strategies, highlighting the sectors best positioned to benefit from targeted interventions, such as those where Ukrainian businesswomen can contribute to strengthening Poland’s industrial landscape. These include industries requiring specialised managerial expertise, innovation-driven sectors that benefit from knowledge spillovers, and areas where skilled labour shortages persist. Overall, the integration of highly skilled Ukrainian women in Poland could enhance both economic diversification and competitiveness, positioning Poland as a regional leader in leveraging skilled migration for industrial innovation and long-term growth.

## Literature review

This section introduces an industry-centred and gender-sensitive analytical framework for understanding skills’ utilisation in host economies, which builds on three strands of literature: (1) migrant skill matching and complementarities in specialised industries [cf. 12,13], (2) diversification via relatedness and adjacent industry upgrading [cf. 14–16], and (3) gendered constraints shaping labour-market allocation and the realised allocation of skills [cf. 17–19]. It is organised around three complementary mechanisms:

Mechanism 1 (M1): Matching & Complementarities – potentially stronger absorption where pre-war skills of Ukrainian women managers coincide with host-country specialisations, enabling within-path upgrading and productivity gains.
Mechanism 2 (M2): Diversification via Relatedness – entry points where those skills are proximate to, but not yet embedded in, the host industrial portfolio, supporting adjacent diversification and industrial upgrading.
Mechanism 3 (M3): Gendered Sectoral Constraints – institutional, organisational, and social barriers (e.g., regulated-profession licensing, care regimes, recognition hurdles, discrimination) that shape realised utilisation of skills.

### Integration of high-skilled migrants, and the gender dimension

International migration profoundly influences local labour market dynamics, industrial specialisation, and long-term economic development [11]. The effective economic integration of migrant workers depends on their engagement in key aspects of the host country, including labour market participation, as noted by Ager and Strang [4]. Within this process, the recognition of qualifications, licensing requirements, and the broader economic and policy context are critical determinants that shape integration outcomes [20]. Access to opportunities and sectoral conditions are crucial, as the industries employing migrants strongly influence their economic outcomes and vulnerability to exploitation or deskilling [21].

Importantly, economic migrants and refugees often experience different labour market trajectories due to variations in legal status, migration motives, and expected length of stay [22]. Both groups can face significant barriers to entry, frequently relying on temporary, low-paid, or lower-skilled jobs as an initial step into employment [21,23]. Refugee women often experience a “double disadvantage”, which can be further exacerbated by factors such as race, ethnicity, age, disability, sexual orientation, or socioeconomic status, resulting in a “triple disadvantage” [18]. How these layered disadvantages translate into labour-market outcomes is highly context-dependent, shaped by host-country institutions (e.g., credential recognition, cultural proximity, and local labour-market structures), which was visible during Europe’s post-2015 refugee inflow [24]. In Turkey, for example, Syrian refugee men exhibit the smallest employment gaps in manufacturing, while refugee women fare relatively better in agriculture, underscoring how cultural proximity, pre-existing networks, and a large informal sector can channel integration pathways differently from EU settings [25]. Comparative evidence across OECD countries likewise shows substantial heterogeneity in refugee–migrant labour-market gaps over time and across host contexts, reflecting differences in institutions and recognition regimes [26].

Scholars have long studied skilled migration [17,27,28], but definitions of “highly skilled migrant” vary, including formal education, professional experience, and industry-specific expertise [29]. While tertiary education is a common proxy for skill, a substantial portion of highly skilled migrants derive their expertise from work experience rather than formal credentials. Focusing solely on academic qualifications may therefore underestimate the economic potential of skilled migrants, particularly in countries like Poland where many Ukrainian women bring valuable industry experience despite facing formal credentialing barriers [19].

Since the 1980s, migration has become increasingly feminised, with women representing a growing share of skilled migrants [30], however, highly skilled migrant women remain underrepresented in research [31]. Migration studies have traditionally focused on male-dominated sectors, leaving gender-specific challenges in professional and managerial roles underexplored [17]. Notably, many highly skilled migrant women experience overqualification and deskilling, highlighting the need for policies that address the intersection of gender and migrant status [32]. Evidence also shows that the realised utilisation of skills is shaped by institutional, organisational, and social arrangements, such as licensing requirements in regulated professions, recognition of qualifications, care-system design, exposure to discrimination, and the gendered composition of sectors [cf. 33–36]. Recognising these challenges is crucial, as integrating a gender perspective into migration policies not only promotes equality but also enhances economic outcomes [37]. However, gender-blind approaches often obscure these dynamics, limiting effective policy responses [38]. Recognising and addressing these barriers is crucial for designing effective policies that not only support refugee women’s employment but also enable host economies to capitalise on their skills.

### Skilled migrants, innovation, and economic growth

Economic growth relies on the continuous development of human capital, knowledge transfer, and technological innovation, all of which are shaped by the integration of a skilled labour force [39,40]. In this context, highly skilled migrants contribute significantly to productivity and industrial dynamism in host economies by introducing new skills, fostering knowledge diffusion, and increasing workforce complementarities [41–43]. Both high- and low-skilled migrants contribute significantly to the host labour market by filling roles that natives may be unqualified for or reluctant to take [1]. Importantly, interactions between skilled migrants and local workers amplify productivity and knowledge dissemination in host countries [6]. Consequently, the influx of skilled workers enhances firm and sector performance by leveraging complementarities with existing capabilities, ultimately boosting productivity [44].

Skilled labour mobility is a key driver of knowledge transfer, leading to productivity improvements [45] and the diffusion of knowledge across regions and sectors [46]. Migrants with industry-specific skills play a crucial role in the emergence of new industries, and their influx supports regional skill enhancement and diversification [47]. Industry-specific knowledge and managerial skills significantly impact firm performance by fostering innovation, competitiveness, and effective problem solving [48]. Such expertise drives productivity growth, especially among high-technology firms [49]. Management team diversity in educational and sectoral backgrounds enhances firms’ performance by fostering knowledge spillovers and driving targeted innovation strategies, resulting in a more innovative product portfolio [50,51].

The debate over whether economic diversity or specialisation drives industrial transformation remains central to migration and labour market research. Specialisation-based arguments suggest that firms benefit from clustering around existing industries, as geographic proximity enhances knowledge sharing and productivity growth [12]. From this perspective, skilled migrants can accelerate technological advances in specialised industries by introducing complementary skills and managerial capabilities [46], aligning with M1 in our framework. Conversely, diversity-based perspectives emphasise inter-industry linkages as drivers of innovation, whereby skilled migrants’ diverse knowledge cross-pollinates sectors, consistent with M2 in our framework. Taken together, the classic accounts associated with Marshall [12] (specialisation) and Jacobs [14] (diversity) provide a concise conceptual bridge between within-path upgrading and related diversification, clarifying how migrant expertise can both reinforce established industries and generate spillovers into technologically or organisationally related activities.

### Ukrainian migration, gender dynamics, and market integration in Poland

In Ukraine’s pre-war economy, men dominated high-level managerial and directorial roles [52]. Nevertheless, the 2017–2020 period saw progress in women’s representation in business leadership [53]. In recent decades, rising emigration driven by political instability, economic challenges, and cultural shifts, has caused brain drain and skill mismatches in the Ukrainian workforce [54,55]. Importantly, these emigration patterns were not uniform across destinations. In parts of Southern Europe (e.g., Italy), labour migration from Ukraine was highly feminised, driven by sectoral demand in domestic and care work. While many of the women engaged in these activities did not have tertiary qualifications, a considerable share were skilled or highly educated professionals whose qualifications were under-recognised and thus channelled into low-paid, gendered labour niches [56]. This situation illustrates Kofman’s [17] observation on the invisibility of skilled female migrants and Bircan and Yılmaz’s [38] critique of gender-blind migration theories that overlook structural constraints.

By contrast, migration from Ukraine to Poland until 2022 was predominantly male, shaped by structural demand in temporary, blue-collar sectors such as construction, agriculture and transport [57]. Ukrainian women in Poland were more often employed in long-term roles in domestic services, care work, and certain agricultural jobs, with their share growing gradually over time [58]. These contrasting patterns highlight how gendered migration flows are closely tied to the sectoral composition of labour markets in different destination countries.

In recent years, however, a growing presence of Ukrainian migrants in hospitality, retail, and entrepreneurship indicates a broader shift in Poland’s migration landscape. The outbreak of war in 2022 profoundly reshaped previous migration patterns. The Ukrainian refugee inflow was predominantly female, with 80% of arrivals being women aged 30–44 [2], and included a significant share of highly educated professionals – over 50% held higher education degrees and had diverse professional backgrounds [59]. Despite their qualifications, many encountered significant barriers to labour market integration, including language obstacles, restrictions on accessing regulated professions, and challenges in obtaining long-term residency [2], leading many to accept jobs below their skill level [60]. In the case of Ukrainian refugee women, multiple structural barriers can intersect to hinder effective labour-market integration. Aigner et al. [19] show that even women with substantial professional experience face significant deskilling risks when such barriers remain unaddressed. These findings reinforce earlier evidence on underemployment and “brain waste” among highly skilled migrants [61]. In Poland, caregiving responsibilities and limited access to childcare services present additional challenges for refugee women with children [62], further constraining their ability to re-enter professional roles and advance in the labour market. Beyond these individual and structural barriers, broader social and cultural integration conditions, such as workplace inclusivity, access to affordable and adequate housing, and the availability of reliable childcare, are also critical to sustaining long-term labour market participation. Comparative evidence [19] from Austria and Poland, shows that the absence of such enabling conditions can exacerbate deskilling risks for highly skilled refugee women, underscoring the need for integration strategies that address both economic and social dimensions.

These varied experiences underscore the complexities of labour market integration of Ukrainian women refugees in Poland [63,64], emphasising the need for targeted policies that effectively harness refugees’ skills and enhance their contributions to host economies. Incorporating a gender-sensitive perspective into these policies would address both immediate barriers to employment and the long-term underutilisation of refugee women’s human capital, while also unlocking opportunities to match their skills with industries in which Poland is already specialised and to leverage their expertise for diversification into related industries.

Synthesising these strands of literature, our framework builds on the premise that the inflow of highly skilled Ukrainian women may interact with host-industry structures through two canonical pathways: matching and complementarities within existing specialisations (M1), and relatedness-based entry into adjacent sectors (M2). However, the realised utilisation of these skills is contingent upon gendered sectoral constraints (M3), which can systematically dampen the returns to M1 and M2 even where sectoral fit or capability proximity is high. We focus analytically on M1 and M2 as the measurable industry pathways commonly operationalised in the specialisation/diversity literature [12–14], while recognising that gendered sectoral constraints (M3) fundamentally shape how, and to what extent, these pathways translate into actual labour-market outcomes.

## Methodology

### Data

This study employs firm-level data from Bureau van Dijk’s Orbis global database [65], a comprehensive database that provides coverage for over 108 million firms worldwide (including more than 20 million are situated across Europe, with approximately 2.5 million employing 10 or more individuals), offering key variables such as industry classification, revenue, employment size, and gender composition in managerial roles, geographic location, among others. The database allows for cross-country comparisons in firms’ specialisation patterns, making it particularly suitable for studying sectoral and labour market structures. Orbis database has been extensively validated in the literature as a reliable dataset for analysing firm-level labour market characteristics. Studies comparing Orbis with official statistics confirm that it captures a significant portion of formal sector employment. Farole et al. [66] found that Orbis covers approximately 73% of total firms in Spain, Poland, Italy, and Romania, with national-level representation ranging from 58% to 89%. Zalas and Drążkowski [67] utilised Orbis database to examine labour share evolution in Poland, demonstrating its applicability in employment analysis.

To align with the research objectives, we collected information on firms’ industries (categorised by NAICS4) and their operating locations (classified by NUTS1), employment levels, and managerial gender composition. We focused on European countries that, up to the cutoff date in June 2023 and according to the UNHCR [68], had a recorded Ukrainian refugee population of 50,000 or more. The 20 selected countries, listed in descending order of refugee intake, were Germany, Poland, the Czech Republic, the United Kingdom, Spain, Italy, Bulgaria, Romania, Moldova, Slovakia, the Netherlands, Austria, Ireland, Lithuania, France, Belgium, Switzerland, Portugal, Finland, and Hungary. We also included Ukraine; however, because of the absence of a NUTS1 classification in Ukraine, firms were grouped by their respective Oblasts into three general areas: West, Central/North, and East/South.

Following the methodology of Kalemli-Ozcan et al. [69], to ensure data quality and representativeness, we applied a structured data-cleaning process by removing firms with incomplete or inconsistent records, and duplicate entries at the NUTS1 regional level. Consistent with studies by Farole et al. [66], Bajgar et al. [70], Bussolo et al. [71], and others, we addressed the issue of underrepresentation of small firms by selecting firms that reported at least 10 employees, location (NUTS1), industry (NAICS4), and managerial gender composition for at least one year during 2017–2021. The multi-year requirement reduces volatility from one-off firm records and ensures comparability across countries.

The final dataset comprises 1,515,295 firms employing approximately 145 million workers across the selected European economies. Within this dataset, 8.3 million managers were identified, of whom 2.2 million were women (26.6%). In line with OECD/ISCO conventions, we operationalise “highly skilled” as managerial roles. In our data, pre-war Ukrainian women’s managerial presence by industry serves as a proxy for industry-specific expertise likely to be transferable to Poland.

A comparison with official national statistics for Poland and Ukraine, before the war commenced, confirms the strong representativeness of the Orbis database for firms with 10 or more employees. According to the European Commission [72], Ukraine had approximately 64,668 enterprises classified as small (10–49 employees), medium (50–249 employees), and large (250 + employees), employing 4.68 million workers. The Orbis dataset for Ukraine includes 74,886 firms, closely aligning with these figures, while capturing a slightly larger workforce of 5.7 million employees. Similarly, in Poland, official statistics indicate that there were 67,679 enterprises with at least 10 employees, employing 5.6 million workers [73]. The Orbis dataset includes 52,316 such firms with a total workforce of 5.6 million employees. These small discrepancies may be attributed to variations in classification methodologies, updates in firm registries over time, COVID-related distortions, and the pre-war cutoff period of the Orbis dataset (2017–2021), which may capture structural labour market shifts not reflected in earlier national reports. Furthermore, the Orbis data for Ukraine includes approximately 79 thousand workers in managerial positions, out of which approximately 19 thousands are women; while for Poland, it includes approximately 186 thousands workers in managerial positions, of which 54 thousands were occupied by women before the war.

### Network construction

We constructed a network that encapsulated the statistical relationships – or ‘relatedness’ – of industries across various regions in the 20 European countries that have hosted the largest populations of Ukrainian refugees, as well as in Ukraine itself. This network is referred to as the ‘benchmark industry network’, which serves as a structural reference point against which Poland’s and Ukraine’s industrial profiles can be compared. Network construction draws upon the conceptual frameworks and methodologies within the economic complexity literature [74,75]. It also adheres to the principle of ‘relatedness’ [15], applying it to the level of industry-regional employment. This network design allows for a comparative examination of how Poland and Ukraine are positioned and specialised in distinct industries compared to the benchmark industry network. By embedding Ukrainian and Polish data into a pan-European benchmark, one can identify industries where skills brought by Ukrainian women managers could be most effectively matched to Poland’s existing specialisations.

Influential research introduced the ‘Product Space’ concept, measuring product relatedness through a proximity indicator based on co-exportation frequencies between countries [76]. This concept was later extended to the industry-regional level, suggesting that regions are more likely to diversify into industries that are technologically or skill-related to their existing ones [16,77]. Such findings indicate that regional capabilities, including skills, play a crucial role in developing new industries. This is particularly relevant for refugee integration, as it implies that skills carried by newcomers can facilitate diversification into related industries, even if these industries are not yet well established or specialised in the host economy.

Furthermore, geographic clustering of firms is driven by three Marshallian mechanisms: the sharing of goods, labour, and ideas, each playing a substantial role in influencing co-location patterns [78]. These mechanism underpin our interpretation of “relatedness” not only as a technological or input-output relationship but also as a human capital and knowledge-spillover relationship.

We analysed the collocation patterns of employment across various NUTS1 regions in selected European economies to quantify industry interconnections. An industry is likely to benefit not only by direct shocks -in the form of knowledge or other – to itself but also by shocks benefiting other co-located industries [79] or industries in locations geographically closer [80,81]. This has significant implications for migration studies; a positive shock in the form of new knowledge introduced into a specific industry in Poland could have cascading benefits. This could enhance not only the output of that particular industry but also that of other interconnected industries.

In this context, a measure of geographic specialisation: the Location Quotient [LQ], offers a nuanced comprehension of industries that possess a high degree of specialisation in specific regions.


$${LQ}_{il}=\frac{X_{il}/X_{l}}{\sum_{l}^{L}X_{il}/\sum_{l}^{L}X_{l}},$$

where ‘*X’* is employment, *‘i’* represents the industry, ‘*l’* the location under analysis and ‘*L’* the number of total locations.

This measure aids in evaluating an industry’s relative significance in a particular regional or national economy relative to its importance across all regions and countries under study. An LQ value exceeding one indicates that a region or country exhibits a specialised pattern in that industry.

The industry network quantifies the likelihood that two industries will co-locate in a NUTS 1 region within the selected European host countries, conditional on both exhibiting and LQ > 1. Higher probability values signify closer interindustry proximity. These proximity values capture diverse forms of interlinkages, which can manifest in various channels such as supply chain dependencies or other knowledge-sharing arrangements.

Industry co-location was initially driven by value chain partnerships, where firms benefited from proximity to suppliers and customers. Recent evidence, however, highlights a shift towards skill sharing as the primary factor influencing co-location, especially within the service sector and knowledge-intensive business services. These evolving dynamics suggest that future industry locations will increasingly depend on the availability and mobility of specialised skills [82].

In summary, we employed network analysis to discern inter-industry proximities within host countries, as defined by our benchmark industry network. A nuanced understanding of these interrelationships is essential to assess how Ukrainian women’s managerial skills can most effectively benefit the economies of host countries. Identifying the main industries, especially those that align with the expertise brought into the country by highly skilled Ukrainian refugees, could facilitate the formulation of strategies to bolster existing industries at regional and national levels.

### The benchmark industry network

**Fig 1:**
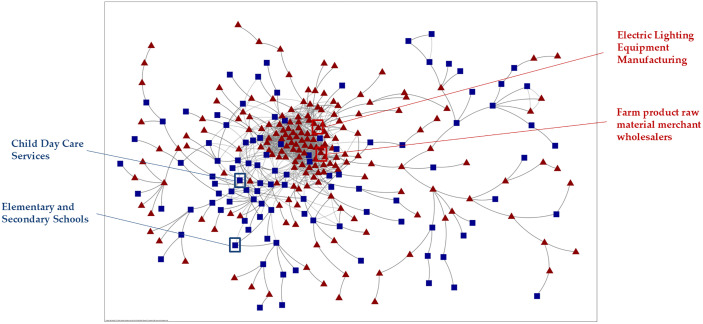
The benchmark industry network. Source: Authors’ elaboration using Orbis global database. Note: In the network representation, nodes symbolise individual industries, and edges denote the proximity or relatedness between pairs of industries in countries hosting the largest number of Ukrainian refugees. Square nodes (104) represent industries where the presence of women managers exceeds the average of the host countries (26.6%), whereas triangular nodes (184) denote industries below that threshold (lower representation of women managers). For graphical clarity, the network displays only 1,364 strongest connections (edges) for 288 industries (nodes), while ensuring to have a connected network (minimum spanning tree using Kruskal’s algorithm) and sparse visualisation without leaving aside any essential industry connections (industries with proximities higher than 0.5).

The benchmark industry network delineates the collocation probability between various pairs of industries across major European nations hosting Ukrainian refugees. This network offers valuable insights into the relative presence or absence of specific industries in each country included in our study. In this study, we use it to contrast the industrial structures of Ukraine and Poland, thereby establishing a reference framework for assessing how refugee women’s skills can be matched to existing specialisations or leveraged for related diversification (Fig 1).

Within the network, the nodes symbolise the 288 NAICS4 industries in which firms from host economies operated before the onset of the war (out of the 310 NAICS industries under analysis). Conversely, the edges of the network – lines connecting the industries – represent the measured distances between industries, calculated as the inverse probability of co-location within the same geographical area. This measure serves as an index of inter-industry relatedness, which is central for identifying both high-absorption industries (M1) and potential pathways for related diversification (M2).

## Results

The arrival of highly skilled Ukrainian women in Poland presents an unprecedented economic opportunity. Many of them have held senior managerial roles across a range of industries, bringing extensive expertise that can foster knowledge spillovers and innovation in Poland.

We performed a comparative analysis to identify the Polish industries that would benefit the most from including skilled Ukrainian professionals. By comparing the national patterns of industry specialisation for Ukraine and Poland relative to the benchmark industry network, we determined the immediate opportunities for industrial policy in Poland. These patterns primarily correspond to M1 and M2, as they identify industries where existing Polish specialisations can be reinforced by Ukrainian women’s skills and where related but less-developed industries in Poland could gain from targeted integration of this talent, respectively. In other words, these patterns indicate where integration could plausibly complement existing industry dynamics, enhance market competitiveness, and foster related diversification in Poland.

The analysis reveals a gender disparity in managerial participation between the two countries, with Polish industries featuring a higher percentage of women in management roles. Analysis of the Orbis database shows that Polish industries have a higher proportion of women in management teams (27%) compared to Ukrainian industries (23%). World Bank data are consistent with this pattern: in 2019, 28% of firms in Poland had a woman top manager compared to 18% in Ukraine [83,84]. We interpret this difference as evidence of higher baseline representation of women in Polish management, not as direct evidence that newly arrived migrant women will access these roles at the same rate. Migrant status can attenuate or delay entry into leadership, further interacting with gendered barriers. Certainly, integration would depend on easing sector-specific frictions (M3) so that the potential payoffs from matching (M1) and diversification (M2) can be realised.

### Comparative analysis of industrial landscapes in Ukraine and Poland

Poland and Ukraine boast a considerable array of industries, totalling 258 each. However, the proportion of industries achieving notable specialisation (LQ > 1) differs; Ukraine has 142 such industries compared with Poland’s 198. Additionally, the prevalence of women in managerial roles within these industries surpasses the average in host countries, with Ukraine and Poland having 89 and 103 industries, respectively, featuring women management.

Upon closer examination of the specific industries in which women hold managerial roles in Poland and Ukraine, both countries exhibited solid women leadership in the education and healthcare sectors. However, distinct patterns have emerged in other industries. Poland has a significant women managerial presence in fashion-related industries, particularly in apparel manufacturing, aligning with its robust textile and clothing sector. Conversely, Ukraine has demonstrated a notable women managerial presence in its food production industry. These variations underscore the unique industrial landscape shaped by women managers in each country. Poland also tends to lean towards personal care and aesthetic services, such as florists and personal care stores, whereas Ukraine exhibits a more robust women representation in business support services. From the M1 and M2 perspective, these overlaps and divergences point to concrete opportunities that can be activated through targeted matching policies, especially where Ukraine’s women’s expertise is adjacent to or embedded within Poland’s existing industrial structure.

**Fig 2:**
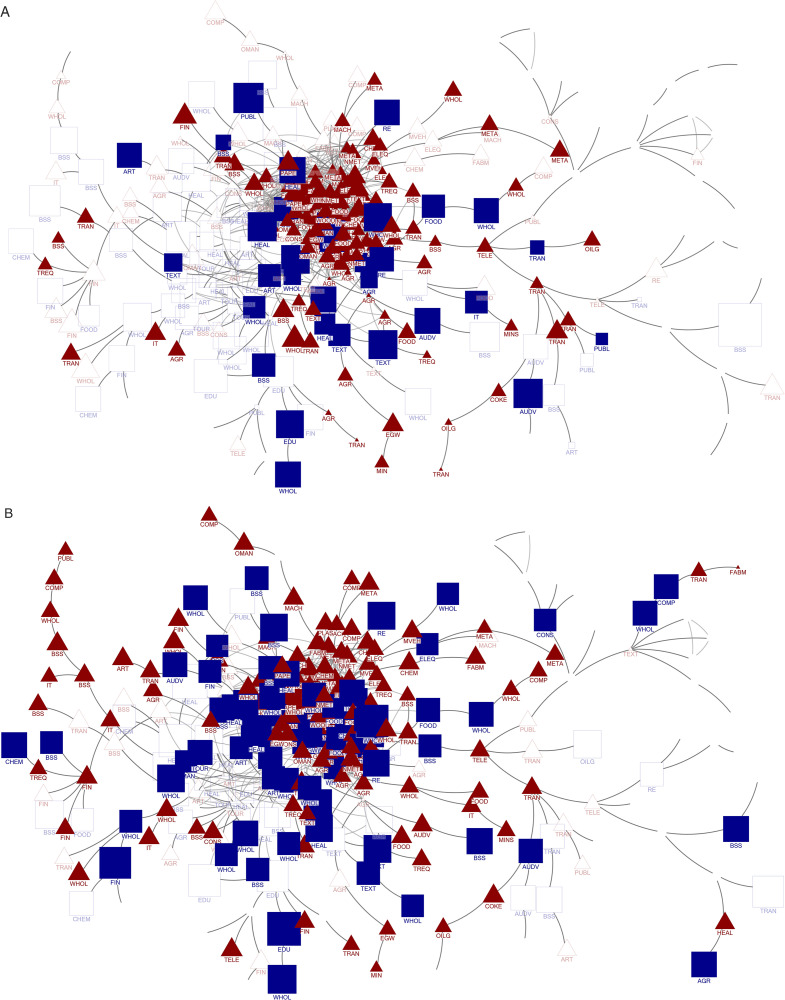
Comparative industry specialisation of Ukraine and Poland in the benchmark industry network. A. Industry landscape for Ukraine. B. Industry landscape for Poland. Source: Authors’ elaboration. Source: Authors’ elaboration. Note: In the network diagrams (Figs 2A and 2B), nodes represent industries and edges show the connections between industries in countries hosting the largest numbers of Ukrainian refugees. The node size shows the share of women managers in Ukraine and Poland's industries. The square nodes indicate industries with more women managers than the host countries' average, whereas the triangular nodes indicate fewer women managers. Empty nodes are industries absent in Ukraine and Poland. Solid nodes denote specialised industries in the country, whereas outlined nodes are less specialised. The node labels identify the economic sector.

The network shown in Fig 2A delineates Ukraine’s industrial landscape. By intersecting industries with a pronounced presence of women managers in Ukraine (89) with those achieving significant specialisation (142), we identified 32 industries (nodes represented by solid squares) in Ukraine as particularly noteworthy. This intersection reflects the unique patterns of women-led industry specialisation in Ukraine compared to the host country’s industry network. Ukrainian firms have demonstrated remarkable performances in these industries, allowing women managerial teams to accumulate extensive knowledge and expertise. Consequently, our analysis focused on revitalising the Polish economy by integrating highly skilled Ukrainian migrant women into these 32 identified industries (Table 1). These industries serve as anchor points for cross-border skill matching and can act as conduits for transferring managerial know-how, further strengthening Poland’s competitive position in related industries.

**Table 1:** Number of industries in Ukraine and Poland: Specialisation patterns and women management participation.

Classification of industries	Ukraine	Poland
Existing industries	258 (90%)	258 (90%)
[1] With specialisation^a^	142 (49%)	198 (69%)
[2] With women in firm’s management team above host countries’ average	89 (31%)	103 (36%)
[1] & [2]	32 (11%)	72 (25%)

Source: Authors’ elaboration. Note: Average women participation in host country firms’ management teams = 26.6%. Total number of NAICS4 industries in which firms from host economies operated before the onset of the war = 288.

^a^Industries with LQ > 1.

We also examined Poland’s industrial landscape, as depicted in the network of Fig 2B. Our analysis identifies 198 industries in which Poland exhibits a relative degree of specialisation compared with other host economies. We observed 60 comparatively underdeveloped industries against the same international benchmarks. Furthermore, in 103 of existing industries, the proportion of women managers exceeded the average in host countries, and 72 of these industries demonstrated a discernible degree of specialisation. From the lens of M2, the existence of these underdeveloped but potentially related industries offers Poland an opening to diversify via relatedness, especially when Ukrainian women bring experience in industries that Poland has yet to fully exploit.

**Fig 3:**
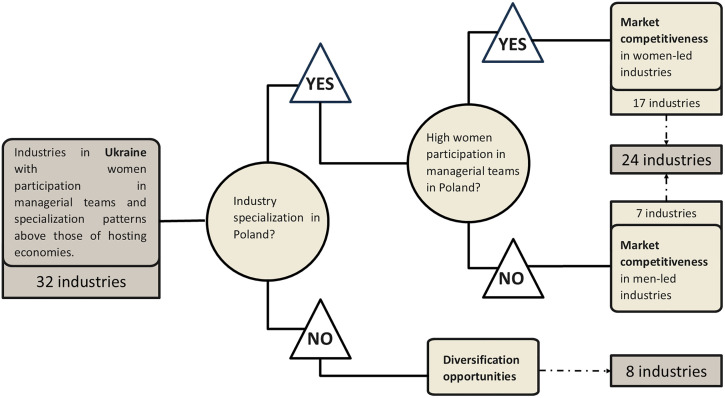
Decision tree for categorising industries with the potential to be shaped by market competitiveness and diversification.

The decision tree in Fig 3 classifies the 32 industries into two categories: (i) industries where Poland already has a degree of specialisation, further divided by whether women’s participation in managerial teams is high or low; and (ii) industries where Poland does not have such specialisation, identified as diversification opportunities. The first category corresponds to M1, while the second aligns with M2.

To evaluate the potential opportunities for Poland, particularly in the context of the 32 industries identified as having a high level of women managerial participation and exhibiting patterns of specialisation in Ukraine relative to the host economies, we adopted a decision tree to categorise these industries based on their potential influence on Polish markets (Fig 3).

Initially, the 32 industries were examined to determine whether they show a degree of specialisation in Poland above that of other host economies. If yes, industries were further divided based on women participation in managerial roles in Poland. If women participation is above that of host economies, the industries were categorised under ‘Market competitiveness in women-led industries’ (17 industries). Otherwise, they were categorised as ‘Market competitiveness in male-led industries’ (7 industries). Industries that did not show a higher degree of specialisation in Poland compared to other host economies were classified as industries with ‘Diversification opportunities’ (8 industries).

This study highlights that 24 industries in Poland, 17 Women-led (Table 2) and 7 Men-led (Table 3), including food manufacturing, textiles, business services, wholesale, real estate, and healthcare, could benefit significantly from the integration of highly skilled Ukrainian women, enhancing market competitiveness via M1. In this scenario, the alignment between Poland’s existing industrial strengths and the skills profile of Ukrainian women creates complementarities that can raise productivity, innovation and firm performance in already specialised industries. This dynamic could shift the focus from traditional migrant-reliant sectors, such as agriculture, construction, and domestic services, as well as the growing gig economy, to areas with higher labour productivity.

**Table 2:** Women-led industries in Poland with the potential to be shaped by market competitiveness (M1).

Description Industry	Economic Sector
Sugar and Confectionery Product Manufacturing	Food products, beverages, and tobacco
Bakeries and Tortilla Manufacturing	
Fabric Mills	Textiles, wearing apparel, leather, and related products
Textile Furnishings Mills	
Apparel Knitting Mills	
Cut and Sew Apparel Manufacturing	
Drugs and Druggists’ Sundries Merchant Wholesalers	Wholesale and retail trade, repair of motor vehicles
Grocery Stores	
Beer, Wine, and Liquor Stores	
Health and Personal Care Stores	
General Merchandise Stores, including Warehouse Clubs and Supercentres	
Lessors of Real Estate	Real estate activities
Activities Related to Real Estate	
Legal Services	Other business sector services
Colleges, Universities, and Professional Schools	Education
Offices of Other Health Practitioners	Human health and social work
Other Ambulatory Health Care Services	

Source: Authors’ elaboration based on industry network comparison between Ukraine and Poland.

**Table 3:** Men-led Industries in Poland with the Potential to be shaped by market competitiveness (M1).

Description Industry	Economic Sector
Other Animal Production	Agriculture, forestry, and fishing
Pulp, Paper, and Paperboard Mills	Paper products and printing
Spring and Wire Product Manufacturing	Fabricated metal products, except machinery and equipment
Radio and Television Broadcasting	Publishing, audio-visual, and broadcasting activities
Other Information Services	IT and other information services
Non-depository Credit Intermediation	Financial and insurance activities
Amusement Parks and Arcades	Arts, entertainment, recreation, and other service activities

Source: Authors’ elaboration based on industry network comparison between Ukraine and Poland.

Table 2 and 3 delineate specific industries poised for growth through increased market competitiveness, contingent upon the successful integration of this proficient group of Ukrainian professionals into the Polish industry landscape.

Specifically, industries in Table 3 represent areas where Ukrainian women had pre-war specialisation in Ukraine but are currently male-led in Poland, suggesting both opportunities for skill transfer and the presence of gender-related barriers.

Seven industries demonstrate a paucity of women managerial participation (Table 3) in Poland, indicating a gender divide that highly skilled Ukrainian women could potentially bridge, which challenges the migration literature’s focus on gender-typical sectors and urges further exploration into women’s experiences in male-dominated fields. It could also suggests a potential double barrier for highly skilled Ukrainian women: entering male-dominated sectors where their expertise could be complementary, yet facing entrenched gender imbalances that limit access to decision-making roles (M3: Gendered Constraints). Addressing these barriers will be crucial to ensure that the competitive potential of these industries is fully realised.

Migration literature has largely concentrated on women entering traditionally female-dominated sectors, often neglecting those who move into male-dominated fields. This gap highlights the need to explore whether migrant women in male-biased industries face similar challenges to their counterparts in female-dominated industries and to examine how these experiences might vary across different national contexts [33].

Economic diversification involves expanding into new economic activities, typically branching into areas closely aligned with existing capabilities [85]. This strategy is vital for economies seeking to grow into new industries effectively. Our analysis highlights eight industries in Poland that present strong opportunities for diversification and could greatly benefit from the specialized skills and experience of Ukrainian women (M2 of Diversification via Relatedness). While they were established in Ukraine and feature a higher women managerial presence, these industries are less prevalent in Poland. In this second scenario, relatedness allows Poland to expand into new industries by leveraging the know-how and managerial experience that Ukrainian women bring, thereby reducing the risks and cost of diversification. Thus, the integration of Ukrainian women may spearhead Poland’s economic expansion into these new areas, as shown in Table 4.

**Table 4:** Industries with the potential for diversification (M2).

Description Industry	Economic Sector	Managerial Team in Poland
Footwear Manufacturing	Textiles, wearing apparel, leather, and related products	Women-led
Other Miscellaneous Store Retailers	Wholesale and retail trade, repair of motor vehicles	
Postal Service	Transport and storage	
Motion Picture and Video Industries	Publishing, audio-visual, and broadcasting activities	
Facilities Support Services	Other business sector services	
Offices of Dentists	Human health and social work	
Administration of Human Resource Programmes	Public administration and defence, compulsory social security	
Performing Arts Companies	Arts, entertainment, recreation, and other service activities	Men-led

Source: Authors’ elaboration based on industry network comparison between Ukraine and Poland.

In sum, the 24 industries where Poland already has a competitive specialisation – 17 women-led and 7 men-led – represent M1, where integration can rapidly strengthen existing market advantages. The 8 diversification opportunities illustrate M2, where Ukrainian women’s expertise could help Poland expand into new but related sectors. The gender disparities observed in the men-led group highlight that even within M1, barriers to women’s entry into managerial roles may persist, warranting targeted integration strategies.

Taken together, the results point to three key findings. First, there is a strong potential for skill matching between Ukrainian refugees and Poland’s existing industrial strengths, but this is uneven across sectors. Second, there are clear opportunities for related diversification, where refugee expertise could extend Poland’s industrial specialisation into adjacent activities. Third, gendered sectoral patterns shape both matching and diversification outcomes, potentially constraining the full utilisation of skilled refugee women’s capabilities. These findings provide a direct empirical bridge to the mechanisms in our analytical framework, highlighting where targeted interventions – such as credential recognition, sector-specific mentorship programmes for women, and diversification incentives – can maximise both economic and integration outcomes.

By linking network analysis with a gender-disaggregated lens, this study moves beyond descriptive mapping to identify actionable pathways where policy can directly influence integration outcomes.

## Discussion

Our findings emphasise the urgency of implementing targeted industrial policies to facilitate the strategic placement of Ukrainian women in industries where their skills align with market needs. Overall, the strategic integration of highly skilled Ukrainian women into the Polish economy – especially in industries where they offer unique expertise – is a significant opportunity for Poland. Leveraging these women’s specialised knowledge and managerial skills can improve the interconnectedness of Poland’s industrial network. By sharing their expertise with local firms and teams across different sectors, they can boost productivity and innovation, consistent with evidence that knowledge transfers through skilled labour mobility raise performance [43]. This reinforces the importance of aligning integration policies with both mechanisms identified: capitalising on complementarities in existing specialisations (M1) while preparing pathways for diversification into related sectors (M2). Furthermore, gendered constraints (M3) emerge as a critical cross-cutting factor. Unlocking many of the high-potential industries for both matching and diversification would require refugee women entering a new labour market and navigating entrenched gender imbalances in managerial roles. This reinforces the importance of integrating a gender-sensitive lens into all stages of labour market policy design, including credential recognition, sectoral upskilling, and leadership pipelines.

Building on our analysis, we propose linking three distinct mechanisms discussed in the migration and industrial policy literature: M1 (Matching and Complementarities), M2 (Diversification via Relatedness), and M3 (Gendered Sectoral Constraints), into a more integrated, industry-specific perspective. We suggest that addressing gender-related structural and social barriers (M3) in isolation is insufficient; rather, these barriers should be considered alongside the other two mechanisms to fully capture the potential of highly skilled migrant women’s expertise. This conceptual linkage offers a way to identify both where migrant women’s skills complement existing specialisations and where they can support diversification into related sectors. In doing so, it outlines a pathway for sectoral integration policies that simultaneously advance gender equity, strengthen a host country’s industry space, and contribute to broader theoretical discussions on migration, gender, and industrial upgrading.

Building on this integrated perspective, our findings underscore the strategic potential of leveraging skilled Ukrainian women’s sectoral experience, but also show that this potential remains conditional on a range of economic and non-economic constraints. These include limited access to specialised Polish language training, lack of streamlined procedures for credential recognition, and gender-based segmentation in the labour market. Additionally, unpaid caregiving responsibilities, childcare shortages, and overrepresentation in informal care roles may further hinder labour market participation. These factors intersect with individual-level differences in prior experience, adaptability, and sectoral fit, underscoring the need for tailored integration strategies. Local authorities and civil society actors play an important complementary role by addressing some of these barriers through access to social services and support programmes. While our study focuses on labour market integration, these broader enablers are essential to unlocking the full potential of refugee women’s contributions.

In the short term, a carefully designed approach to integrate Ukrainian women into sectors that complement Poland’s existing competencies can immediately augment market competitiveness. By swiftly filling skill gaps and management shortages in key industries, Poland can achieve synergistic growth while accelerating the labour market integration of the refugees. This mirrors the emphasis in policy research on rapid integration to maximise refugees’ contributions [4]. For medium and long-term sustainability, our analysis suggests broadening Poland’s economic network to include select untapped industries, by leveraging the underutilised capabilities of highly skilled Ukrainian women. Mobilising their expertise in sectors where Poland currently lacks depth can catalyse diversification into new areas of economic activity, effectively injecting new comparative advantages into the economy, in line with the findings of Bahar and Rapoport [6] and Kancs and Lecca [7]. Fundamentally, embedding these newcomers’ skills into the industrial fabric not only strengthens existing industries but also lays the groundwork for developing new specialisations – fulfilling M1 by reinforcing current capabilities and M2 by strategically branching into related but developing industries. While our findings highlight strategic opportunities for integrating skilled Ukrainian women, it is important to acknowledge that sectoral matching is not uniform. Individual trajectories may vary depending on the nature of prior roles, differences in sectoral standards, and the degree to which previous experience aligns with expectations in the Polish labour market.

Taken together, these patterns point to a dual policy horizon: immediate competitive gains through M1, and targeted diversification via M2 in the medium term. From an economic perspective, the results highlight a greater inclination towards industries that enhance market competitiveness than towards those that foster economic diversification. This suggests that M1 currently offers a broader scope for immediate economic gains, while M2 could represent a targeted, medium-term growth pathway. From a political-economy perspective, the limited range of industries identified for diversification presents a strategic advantage, allowing for focused integration efforts and potentially reducing resistance from veto players, which is often observed in broader policy initiatives. Such seemingly restrictive scope could enable more effective and concentrated integration strategies in specific sectors, deserving further exploration in market integration policy discussions.

In particular, the results suggest a range of policy measures to capitalise on this talent influx and ensure successful integration. First, industry-specific training programs should be developed to bridge skills and certification gaps in high-potential sectors such as specialised manufacturing, IT, and healthcare, equipping highly skilled Ukrainian professionals for success in the Polish market. Additionally, tailored language courses for professionals should be introduced to ensure effective communication in workplace settings, alongside training programs that familiarise migrants with industry norms and work culture in Poland. Such measures would directly operationalise M1 by enabling smoother knowledge matching between Ukrainian professionals and Polish firms. Stronger public-private and academic partnerships can further drive innovation by pairing Ukrainian experts with Polish firms in incubators or R&D projects to address industry challenges and develop new products.

Furthermore, enhancing managerial capabilities through leadership training, mentoring schemes, and management fast-tracks would support Ukrainian women in transitioning into decision-making roles, strengthening corporate governance and productivity and tackling the structural barriers identified under M3. Moreover, support for entrepreneurship is also key – lowering barriers through access to microfinance, startup visas, and business advisory services could encourage refugee women to launch businesses, fostering job creation and market innovation. Notably, greater cultural diversity has been linked to higher entrepreneurship rates [48], suggesting that empowering these women as entrepreneurs could stimulate growth in new market niches. Finally, regulatory reforms should simplify foreign credential recognition, reduce restrictive licensing requirements in professions where Ukrainian women have expertise, and remove unnecessary legal barriers to employing non-nationals in high-skill roles.

The implementation of these measures is crucial to ensure a swift and effective assimilation of this talented demographic into Poland’s workforce. Furthermore, our study highlights the significant cost of inaction: failing to leverage the skills of these refugee women can lead to resource misallocation and welfare losses. In line with the findings of Zetter and Ruaudel [86], failing to utilise their human capital effectively would result in “brain waste,” leading to significant productivity losses for the host economy. Empirical simulations suggest that such untapped potential translates into sizable forgone growth – for example, inadequate integration of refugees has been estimated to cost host countries in terms of lower long-run GDP [7]. Drawing on the findings of Aiyar et al. [3], we argue that decisive policy action is crucial not only to maximise the potential of these professionals but also to foster Poland’s sustained economic growth through the combined operation of Mechanisms 1–3.

Importantly, the findings suggest that policies addressing only general labour market barriers may fall short if they do not account for gendered sectoral dynamics. Interventions that simultaneously target skills matching, industrial diversification, and gender equity are likely to generate the greatest integration and economic spillover benefits.

Our findings advance three debates through an industry-centred, gender-sensitive lens. First, in refugee-integration research, we shift attention from aggregate or low-skill outcomes to industry-specific pathways for highly skilled refugee women, showing where pre-war managerial capabilities can be matched to host-country specialisations (M1) or mobilised for adjacent entry (M2) once gendered frictions are mitigated (M3) [8,17–19,26]. Second, in the economic-complexity/relatedness literature, we operationalise diversification with a cross-country benchmark industry network for economies hosting the largest influx of Ukrainian refugees and use it to identify concrete, proximate job entry points, linking relatedness metrics to the composition of management teams [cf. 15,16,74,76,77]. Third, in work on migration-driven knowledge diffusion and competitiveness, we connect inflows of migrant managers to within-path upgrading and new-activity emergence via documented channels of knowledge transfer and team complementarities [cf. 6,45,51]. Taken together, this proposes an integrated mechanism-based perspective (M1/M2 conditioned by M3) that can travel across host contexts and informs sector-sensitive integration policy.

### Data limitations

While we used data on industries where Ukrainian highly skilled women managers were employed prior to the onset of the war, precise figures on how many of them left the country and their current locations remain uncertain. Some opted for internal migration within Ukraine, whereas others chose various European Union (EU)/non-EU destinations. A substantial portion of them settled in Poland, with a significant number intending to stay permanently [87].

While precise data on the geographical distribution and industry-specific expertise of highly skilled Ukrainian women in Poland is unavailable, this study leverages robust pre-war datasets to deliver actionable insights. By focusing on industries where these women held managerial roles prior to the war, the analysis identifies strategic opportunities to bridge gaps in Poland’s industrial landscape. This approach ensures a nuanced understanding of potential labour market impacts, highlights industries primed to benefit from the integration of skilled migrant women and provides valuable, actionable insights.

The methodology we used in this study highlights the importance of proactive industrial policy interventions to maximise the economic contributions of refugees. Our analysis illustrates how a labour supply shock – specifically, the influx of highly skilled Ukrainian women – can be transformed into an opportunity to strengthen existing capabilities or drive diversification into new industries. This strategic framework provides policymakers with a data-driven tool to develop proactive, evidence-based approaches that enhance both short-term competitiveness and long-term resilience in Poland’s economy. Instead of allowing refugee employment to be determined by ad hoc outcomes, policymakers can strategically direct talent into sectors where it delivers the greatest marginal benefit for both refugees and the host economy.

## Conclusion

This study underscores the strategic imperative of leveraging the expertise of highly skilled Ukrainian businesswomen in Poland’s economy, with a specific focus on the synergies and complementarities between their expertise and Poland’s industry space. The integration of these women into 32 key industries, where they not only hold a competitive edge, but also provide strategic complements to Poland’s existing industry network, serves as a cornerstone for evidence-based policy interventions that effectively blend market-driven solutions with targeted government support. The expected benefits include heightened market competitiveness, enhanced diversification, increased investments, and stimulated innovation across Polish industries, embodying the essence of the industrial policy objectives aimed at fostering a vibrant and robust economy.

Our findings highlight that Poland can benefit from applying these three interrelated mechanisms of integration:

M1 (Matching and Complementarities): enhancing market competitiveness by integrating skilled Ukrainian women into industries where Poland already has a specialisation advantage (e.g., food manufacturing, textiles, healthcare, and business services).
M2 (Diversification via Relatedness): promoting economic diversification by enabling these professionals to contribute to emerging or underdeveloped industries, broadening Poland’s industrial base and stimulating knowledge spillovers.
M3 (Addressing Gendered Sectoral Constraints): ensuring that structural and social barriers do no limit the full utilisation of skills, thereby enabling the other two mechanisms to function effectively.

The economic benefits would be facilitated by forging a landscape conducive to competitive markets, gender-balanced leadership, and innovation. Essential to this endeavour is the creation of an inclusive environment that not only addresses immediate skill shortages, but also harnesses the potential of these adept migrants for overarching economic prosperity. For sustained success, our proposals extend to strategies focused on strengthening the employability of Ukrainian women, through measures such as recognising their qualifications and expertise, targeting upskilling programmes, removing structural and industry-specific barriers to their integration. This holistic approach to skilled migrant integration, informed by our analysis and aligned with strategic industrial policies, could significantly enrich Poland’s economy.

In conclusion, our research highlights the immediate opportunities presented by integrating Ukrainian business women’s expertise in Poland’s industry space, underscoring the importance of targeted market integration policies in European countries. This reinforces the critical role of strategic, evidence-based policymaking in maximising the potential of skilled migrants, advocating for an assertive approach to addressing economic and labour market gaps. This comprehensive strategy for skilled migrant integration, rooted in our network analysis findings and industrial policy considerations, promises to significantly influence the creation of a more innovative, dynamic, and competitive Polish economy.

Building on this study, comparative analyses across different host countries could illuminate how the impact of integrating Ukrainian women varies by context and policy regime. For instance, examining their contributions to market competitiveness and diversification in other European economies would help generalise the findings. Investigating skill mismatches and barriers preventing full utilisation of their talents (in Poland and elsewhere) remains crucial, as does exploring the gender-specific challenges these women face in host labour markets. Addressing these questions will further inform how host countries can turn forced migration into an engine for inclusive economic development.
